# Supplementary material for: Endocytosis regulates TDP-43 toxicity and turnover
Source: Nat Commun. 2017 Dec 12;8:2092. doi: 10.1038/s41467-017-02017-x (PMC5727062; doi:10.1038/s41467-017-02017-x)
Supplement: Supplementary file 1 — Supplementary Information [file 41467_2017_2017_MOESM1_ESM.pdf]

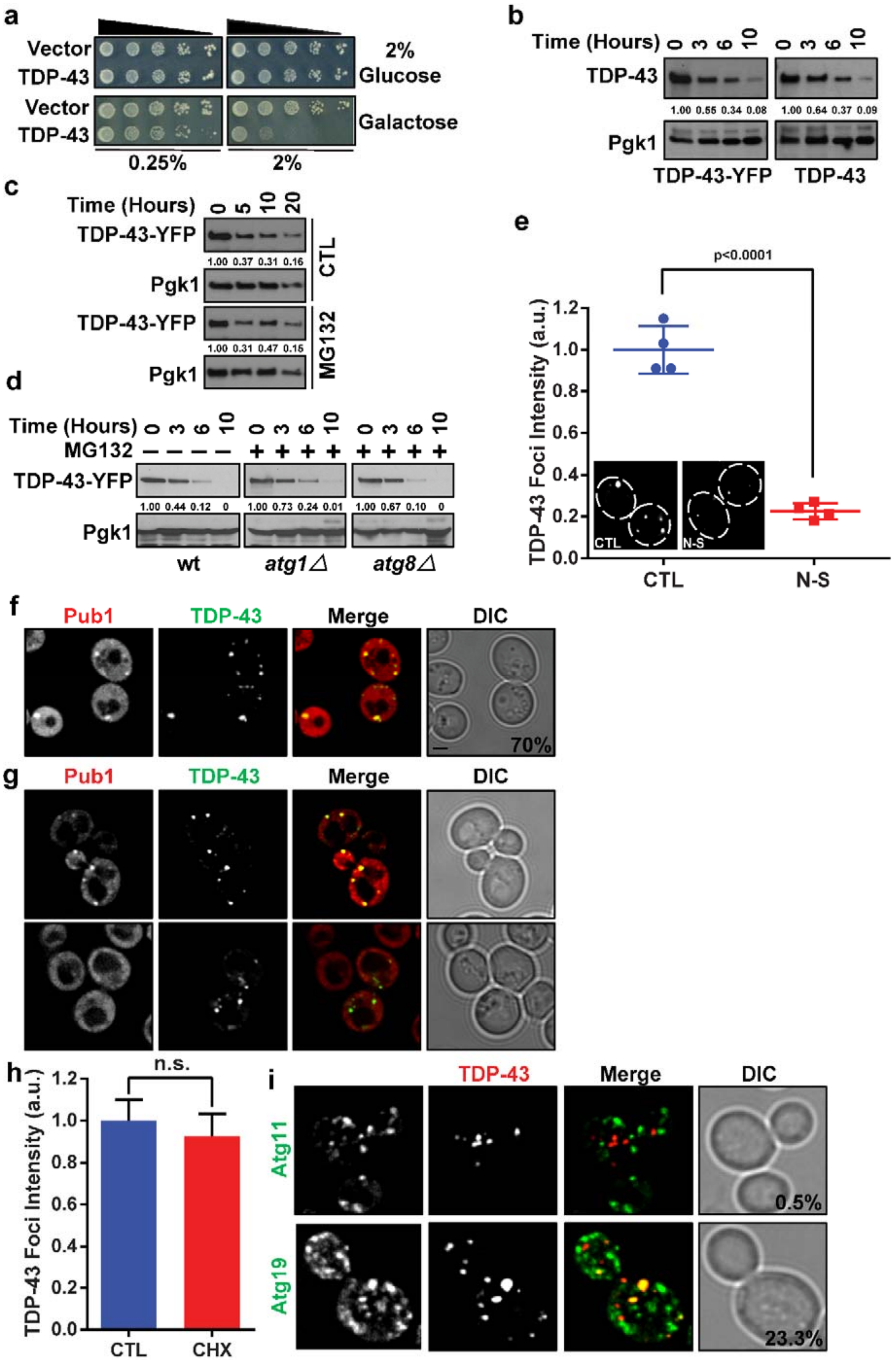
a
Vector
TDP-43
Vector
TDP-43
2%
Glucose
Galactose
0.25%
2%
b
Time (Hours) 0 3 6 10 0 3 6 10
TDP-43
1.00 0.55 0.34 0.08
1.00 0.64 0.37 0.09
Pgk1
TDP-43-YFP
TDP-43
c
Time (Hours) 0 5 10 20
TDP-43-YFP
1.00 0.37 0.31 0.16
Pgk1
CTL
TDP-43-YFP
1.00 0.31 0.47 0.15
Pgk1
MG132
d
Time (Hours) 0 3 6 10 0 3 6 10 0 3 6 10
MG132 – – – – + + + + + + + +
TDP-43-YFP
1.00 0.44 0.12 0
1.00 0.73 0.24 0.01
1.00 0.67 0.10 0
Pgk1
wt
atg1△
atg8△
e
p<0.0001
TDP-43 Foci Intensity (a.u.)
1.2
1.0
0.8
0.6
0.4
0.2
0.0
CTL
N-S
CTL
N-S
f
Pub1
TDP-43
Merge
DIC
70%
g
Pub1
TDP-43
Merge
DIC
h
n.s.
TDP-43 Foci Intensity (a.u.)
1.2
1.0
0.8
0.6
0.4
0.2
0.0
CTL
CHX
i
TDP-43
Merge
DIC
Atg11
0.5%
Atg19
23.3%

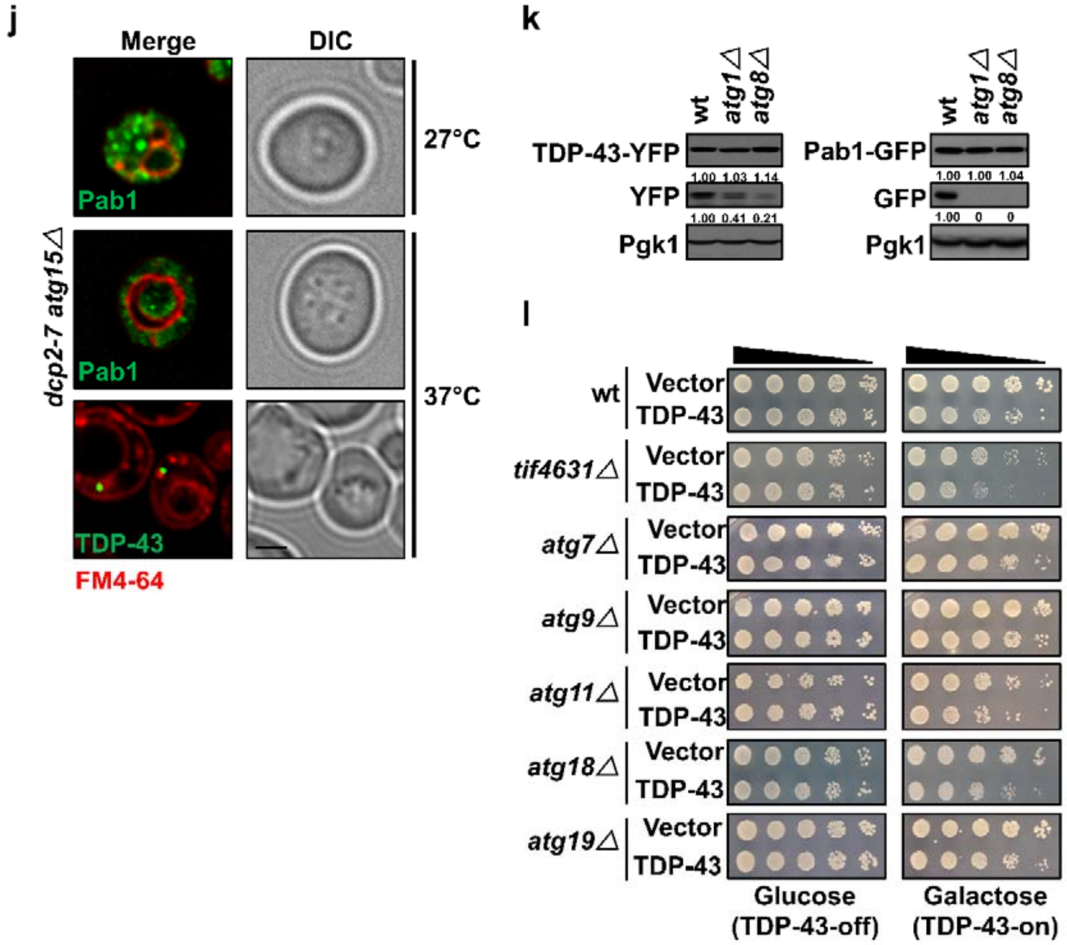

Figure S1: TDP-43 toxicity and turnover in yeast are weakly affected by autophagy and the proteasome. (a) WT yeast transformed with vector or GAL1-regulated TDP-43 under indicated galactose concentration; 0.25% Galactose induction allows better detection of enhancers of TDP-43 toxicity. (b) WT yeast transformed with TDP-43-YFP or TDP-43. TDP-43 turnover rate was examined in mid log as in Fig 1c. (c) MG132-sensitive *pdr5⊿* yeast expressing TDP-43-YFP. Time indicates period following CHX addition (0.2mg/ml) +/- MG132 addition (25ug/ml). TDP-43-YFP turnover rate determined as above (d) Turnover of TDP-43-YFP protein in WT (*pdr5⊿*), *pdr5⊿ atg1⊿* and *pdr5⊿ atg8⊿* strains in mid log phase. Time indicates period following transcriptional shut off +/- MG132 addition (25ug/ml). (e) Transcriptional shut-off of TDP-43-YFP expression in WT cells +/- nitrogen starvation (N-S) for 3 hours. TDP-43 foci intensity was measured and analyzed by via two-tailed Student's t test. Data are shown as mean ± s.e.m. (f) TDP-43-YFP and Pub1-mCh were transformed in WT and examined; % value indicates co-localization of TDP-43 foci with Pub1-mCh foci. Scale bar = 2µm (g, h) As in (f) +/- 0.1mg/ml CHX for 30 minutes - Scale bar = 2µm (g); TDP-43 foci intensity quantified (h). n.s. = no significance. (i) WT yeast transformed with TDP-43-mRuby2, and either Atg19-GFP or Atg11-GFP were examined; % value indicates co-localization of TDP-43 foci with Atg19 or Atg11. Scale bar = 2µm (j) *dcp2-7 atg15⊿* yeast was transformed with either TDP-43-YFP or Pab1-GFP, stained with FM4-64 dye and shifted to 37°C for 1 hour to inactivate Dcp2 prior to imaging. Scale bar = 2µm (k) Indicated strains were transformed with TDP-43-YFP or Pab1-GFP cultured to mid log, and subject to 4-hour N-S. TDP-34 quantified as in Fig 1e. (l) Indicated strains (autophagy mutants) transformed with vector or TDP-43. Serial dilution growth assays were performed as in Fig. 1g.

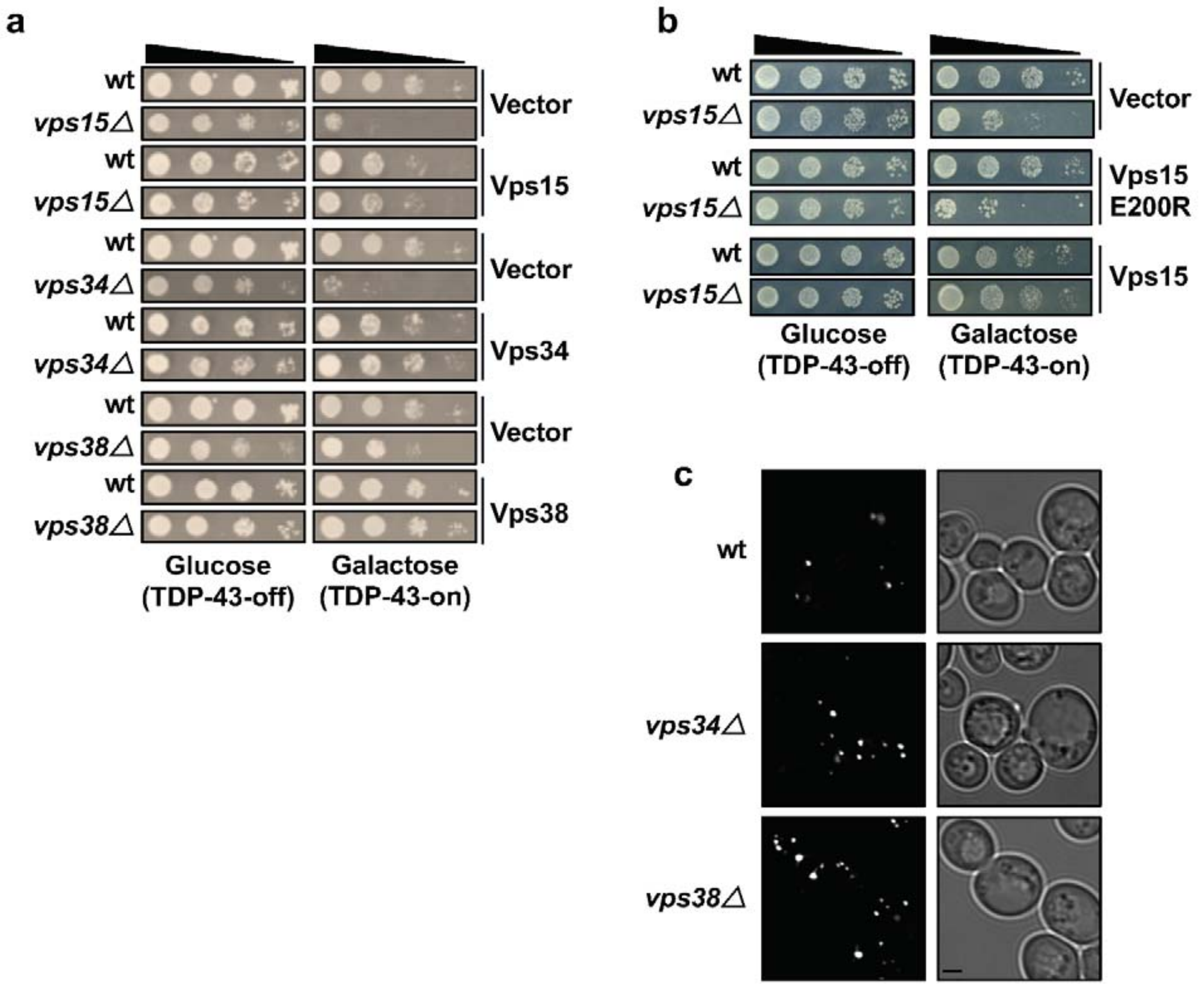

Figure S2: PI3K complex II affects TDP-43 toxicity and foci formation. (a, b) Indicated WT and null strains co-transformed with TDP-43 and either empty vector, Vps15, Vps34 or Vps38 plasmids (A) or empty vector, Vps15 E200R and Vps15 plasmids. Serial dilution growth assay performed as in Fig. 1g. (c) Indicated strains expressing TDP-43-YFP were examined and foci intensity quantified (see Fig. 2c). Scale bar = 2 µm.

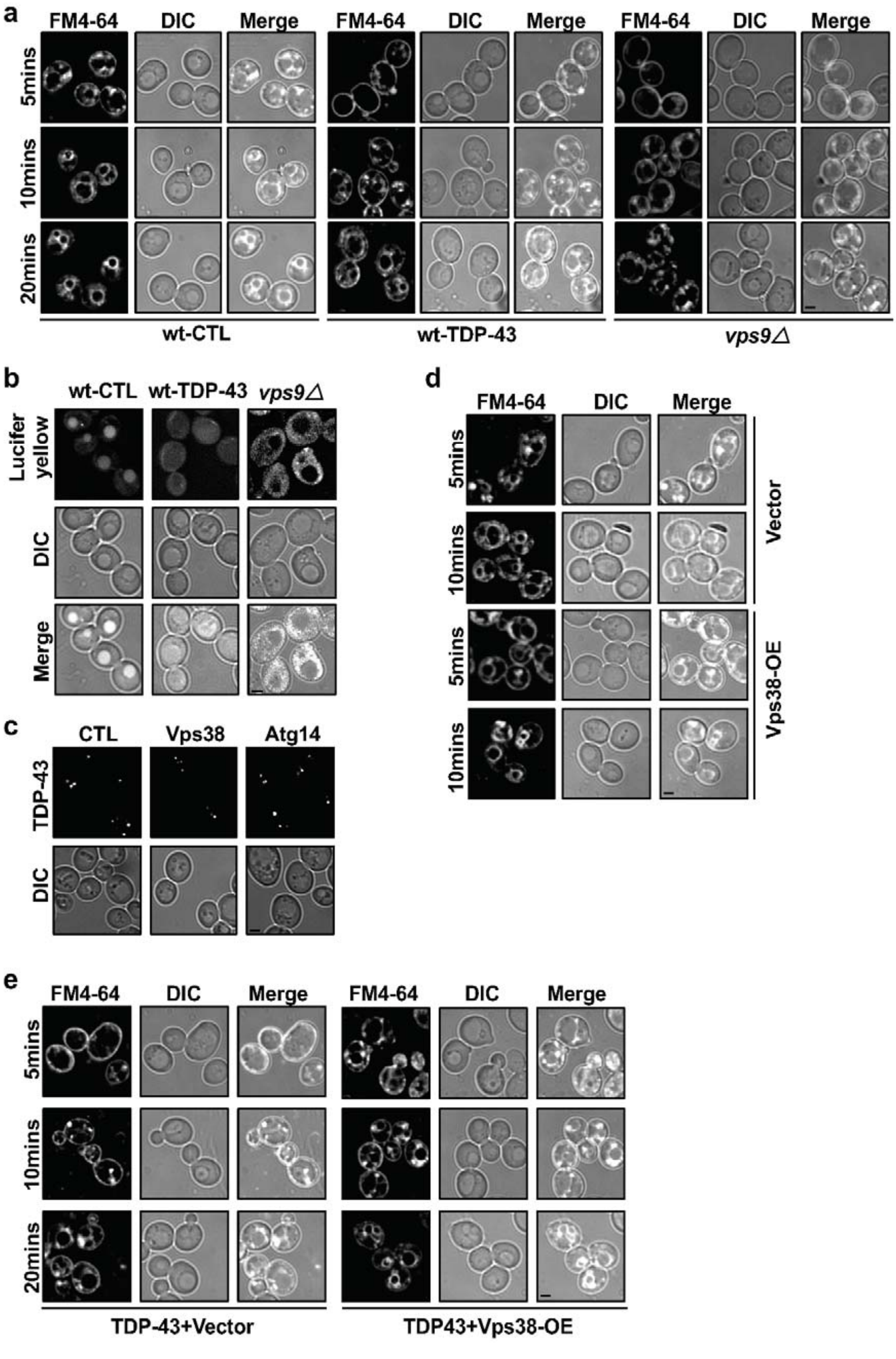

Figure S3: Inhibition of Endocytosis by TDP-43 expression is suppressed by overexpression of Vps38. (a, b) WT cells transformed with vector or TDP-43-YFP, and *vps9Δ* were cultured to mid log phase and stained

with FM4-64 dye (8 uM) for indicated time (a) or lucifer yellow (1 mg/ml) for 30 minutes (b) to reveal endocytosis rate differences. (c) WT co-transformed with TDP-43-YFP and either empty vector, Vps38 or Atg14 multi-copy plasmids, examined under mid log. (d, e) WT cells transformed with TDP-43 and/or multicopy empty vector/Vps38 plasmids were cultured to mid log phase, and stained with FM4-64 dye (8 uM) for indicated time. Scale bar = 2 µm.

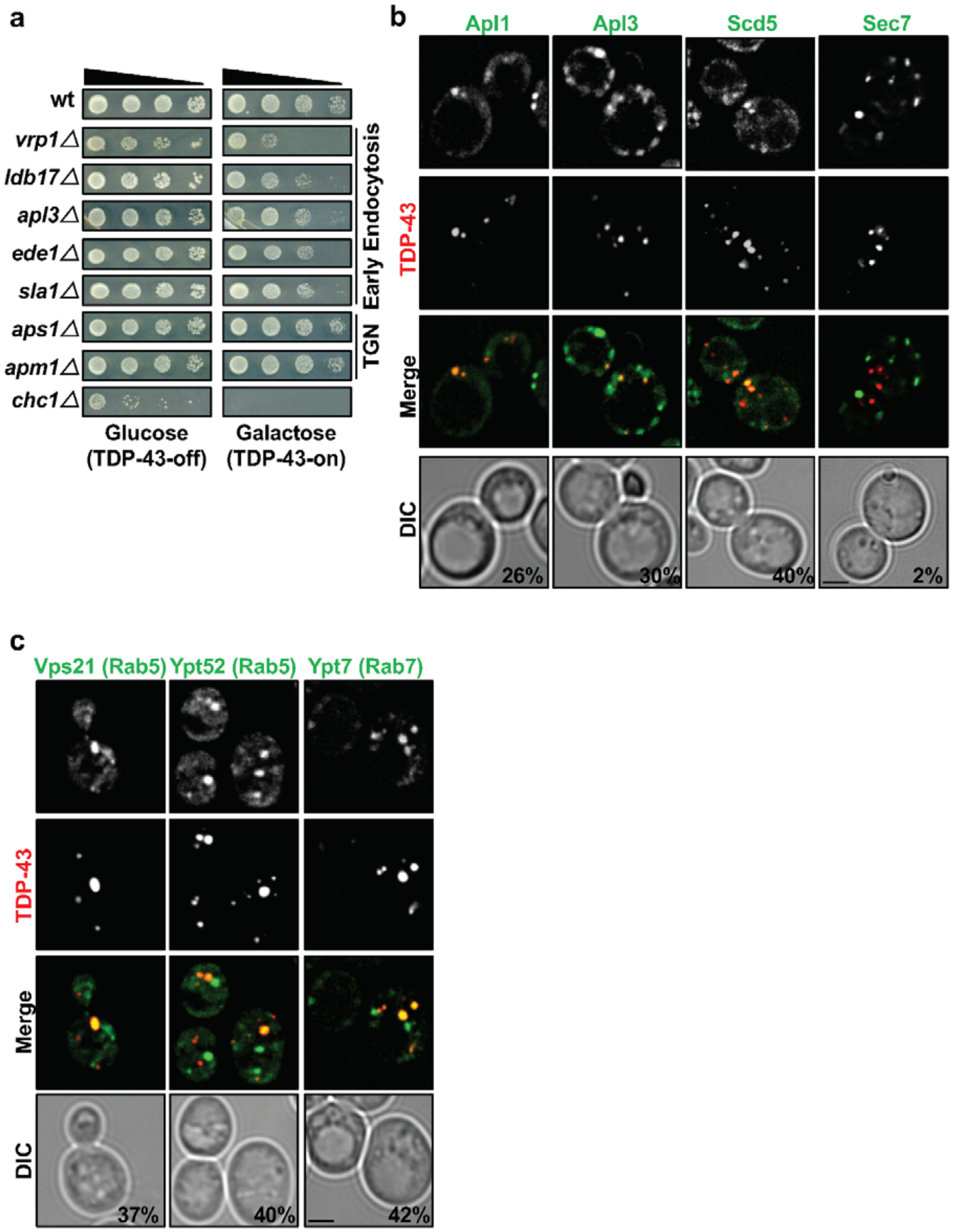

Figure S4: Early Endocytosis factors localize with and modify TDP-43 toxicity. (a) Serial dilution growth assays of WT and null strains (unique endocytosis/Golgi-vacuole trafficking mutants) expressing TDP-43-YFP. (b) Apl1-, Apl3-, Scd5- or Sec7-GFP strains were transformed with TDP-43-mRuby2 and examined. % value indicates co-localization of TDP-43 foci with Apl1, Apl3, Scd5 and Sec7. Scale bar = 2 µm. (c) Vps21-, Ypt52- or Ypt7-GFP strains (Rab5/7 homologs) were transformed with TDP-43-mRuby2 and examined. % value indicates co-localization of TDP-43 foci with Vps21, Ypt52 and Ypt7. Scale bar = 2 µm.

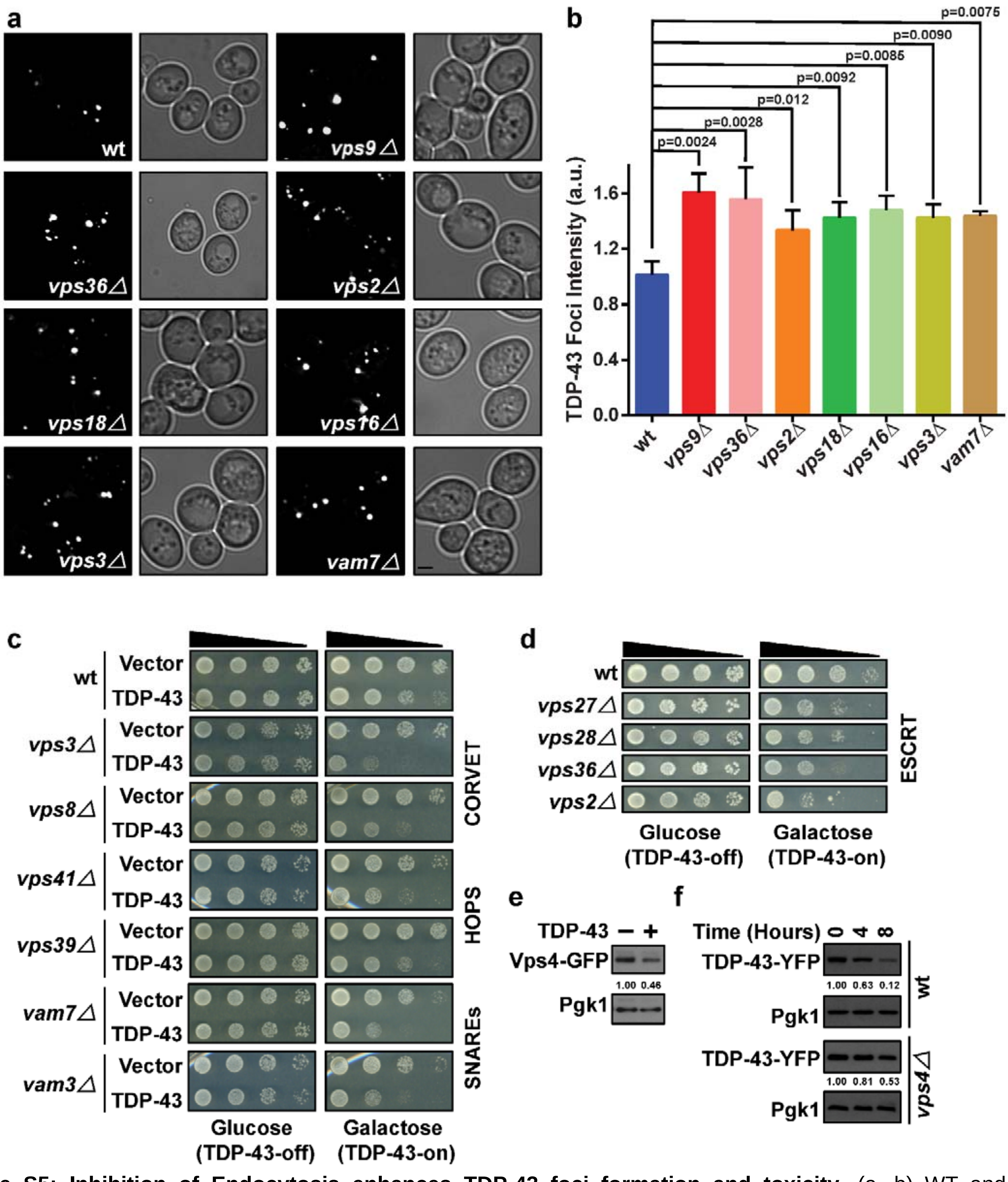

Figure S5: Inhibition of Endocytosis enhances TDP-43 foci formation and toxicity. (a, b) WT and indicated strains were transformed with TDP-43-YFP and examined (a) TDP-43 foci intensity quantified (b). Significance was assessed via one-way ANOVA. Repeated data are shown as mean ± s.e.m. Scale bar = 2 µm. (c, d) Serial dilution growth assays of WT and CORVET, HOPS, SNARE (c) or ESCRT (d) null strains. (e) Vps4-GFP strain was transformed with or without TDP-43, and protein levels were quantified in mid log as in Fig 1e. (f) Turnover of TDP-43 degradation was assessed as in Fig. 1f.

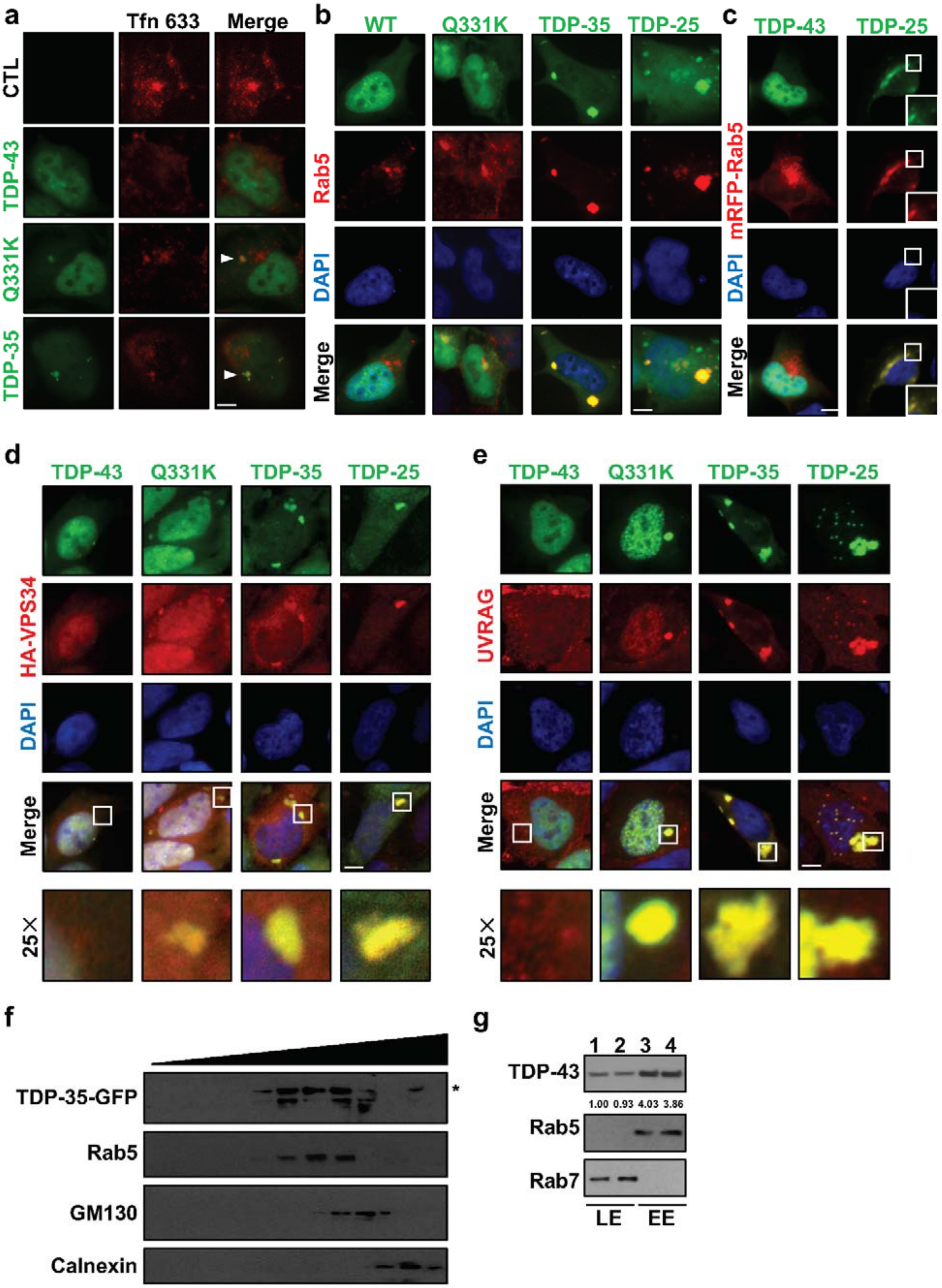

Figure S6: TDP-43 is co-localized with human cell endocytic factors. (a) HEK293A cells transfected with vector, TDP-43-, TDP43 Q331K- and TDP-35-GFP plasmids were incubated with transferrin-633 and uptake

assessed after 15 minutes; quantified in Fig. 5a. Arrowheads indicate TDP-43 and endosome co-localization. (b) HEK293A cells transfected with TDP-43-, TDP43 Q331K-, TDP-35- and TDP25-GFP plasmids were immunostained for endogenous Rab5 and examined. (c) Immunostaining of SH-SY5Y neuroblastoma cells co-transfected with Rab5-mRFP and either TDP-43- or TDP-25-GFP. (d, e) Immunostaining of HEK239A cells transfected with indicated TDP-43-GFP plasmids and HA-VPS34 (d); endogenous UVRAG was detected (e). Scale bar = 5 µm. (f) Fractionation of TDP-35 as in Fig. 5e. Asterisk indicates full-length TDP-35-GFP. (g) Endosome fractionation was performed in TDP-43 transfected HEK293A cells, with western blotting for TDP-43-GFP, and endogenous Rab5 (EE) and Rab7 (LE).

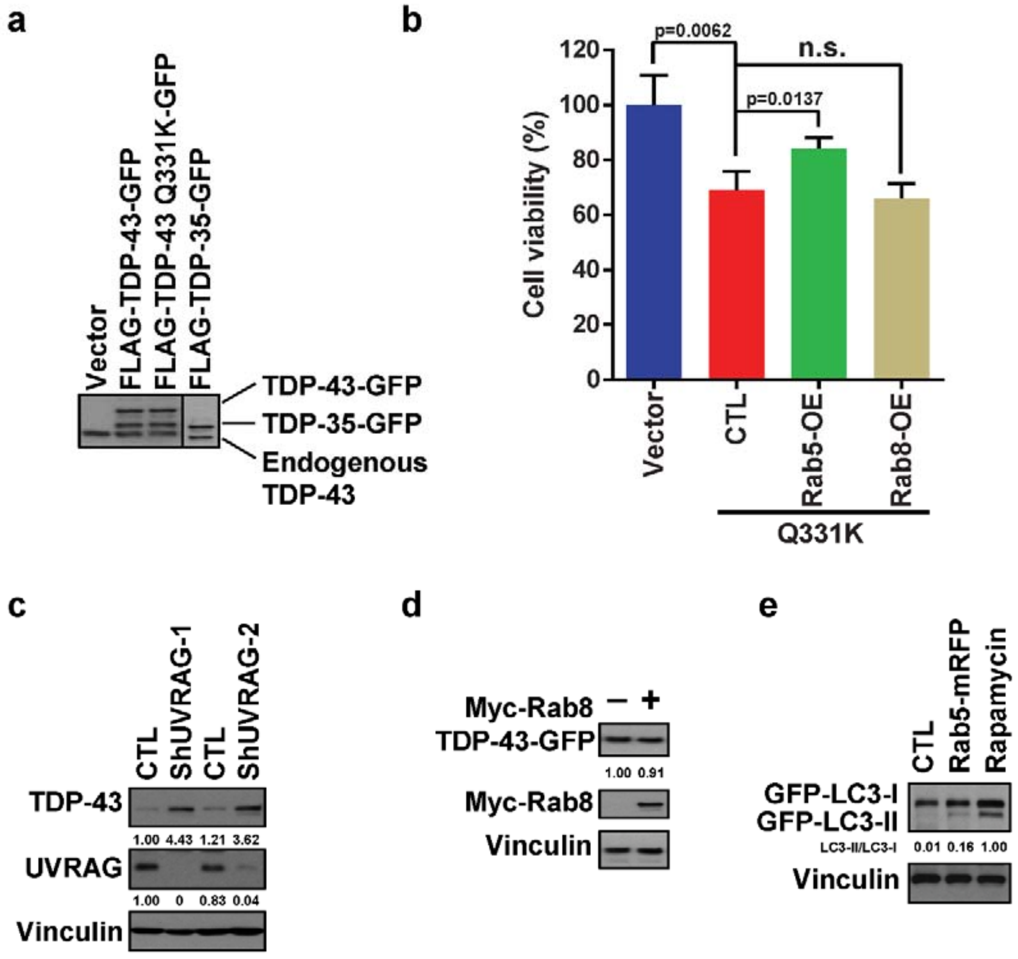

Figure S7: Rab8 over-expression does not promoter TDP-43 turnover or suppress TDP-43 toxicity. (a) HEK293A stable cells expressing integrated vector, TDP-43 WT-, Q331K- or TDP-35-GFP were constructed, and expression level relative to endogenous TDP-43 assessed. (b) Cell viability was tested in HEK293A stable cells expressing vector and Q331K TDP-43-GFP +/- Rab5 or Rab8 over-expression. Significance was assessed via one-way ANOVA. (c) Endogenous TDP-43 expression level was detected and quantified in control (CTL) and UVRAG knock down cells relative to Vinculin levels (loading control). (d) HEK293A cells were -transfected with TDP-43 +/- Rab8 and examined TDP-43 protein levels were quantified as above. (e) HEK293A cells were transfected with GFP-LC3 +/- Rab5, or subject to 50nM rapamycin for 6 hours. LC3-II/LC3-I ratios were calculated and normalized to Vinculin levels.

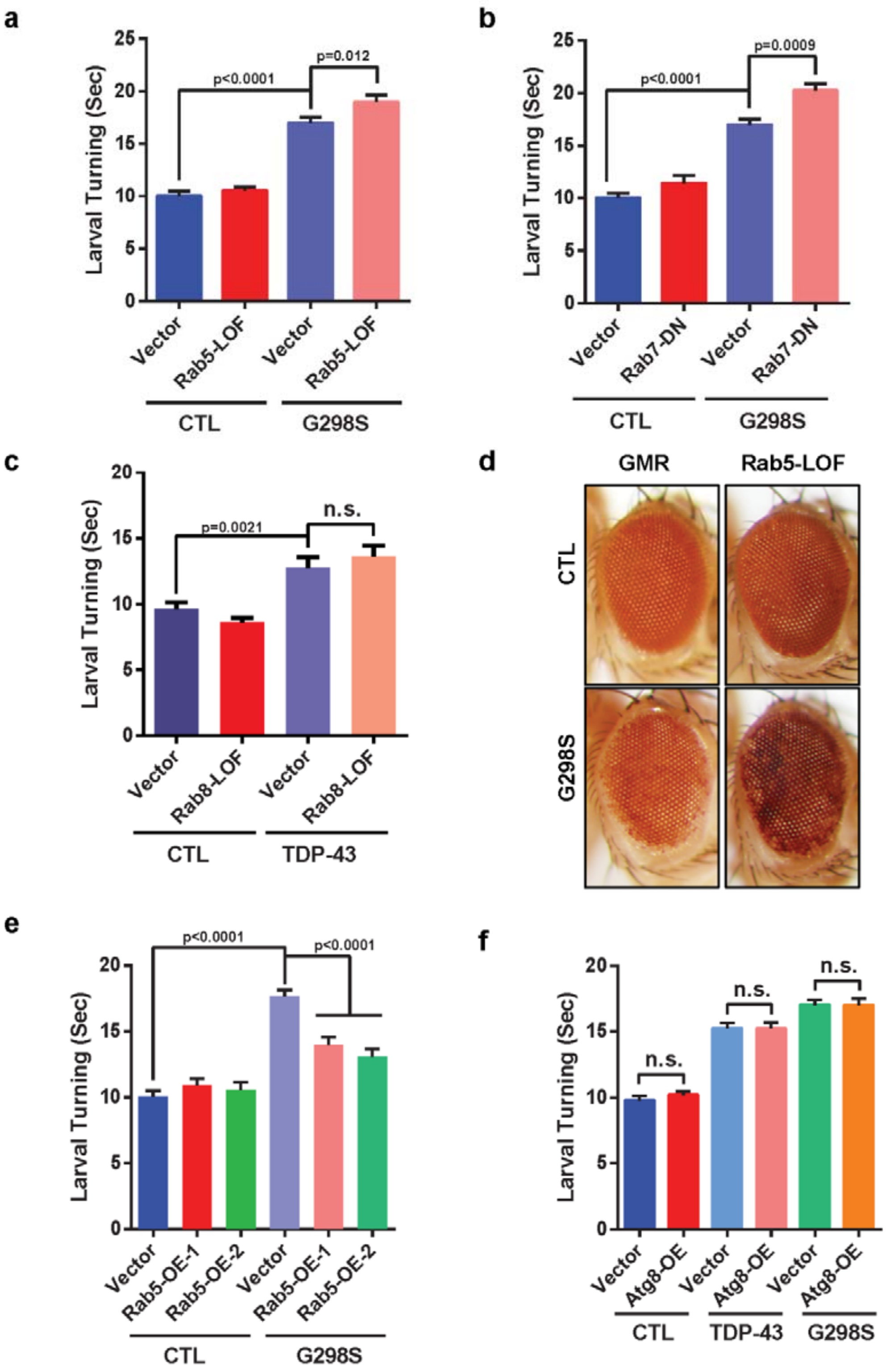

Figure S8: TDP-43-G298S-induced locomotor dysfunction and neurodegeneration is modulated by key endocytic proteins. As in Fig. 7 except: (a) TDP-43 G298S motor neuron expression in Rab5 loss of function (LOF) background. (b) TDP-43 G298S motor neuron expression in Rab7 LOF and dominate negative (DN) background. (c) TDP-43 in Rab8 LOF background. (d) Fly retina neurodegeneration assessed by depigmentation; TDP-43 G298S was expressed using GMR GAL4. Larval turning assay +/- Rab5 overexpression (e) or (f) +/- Atg8. Significance was assessed by two-tailed Student's t test (n.s. = no significance). Data are shown as mean ± s.e.m.

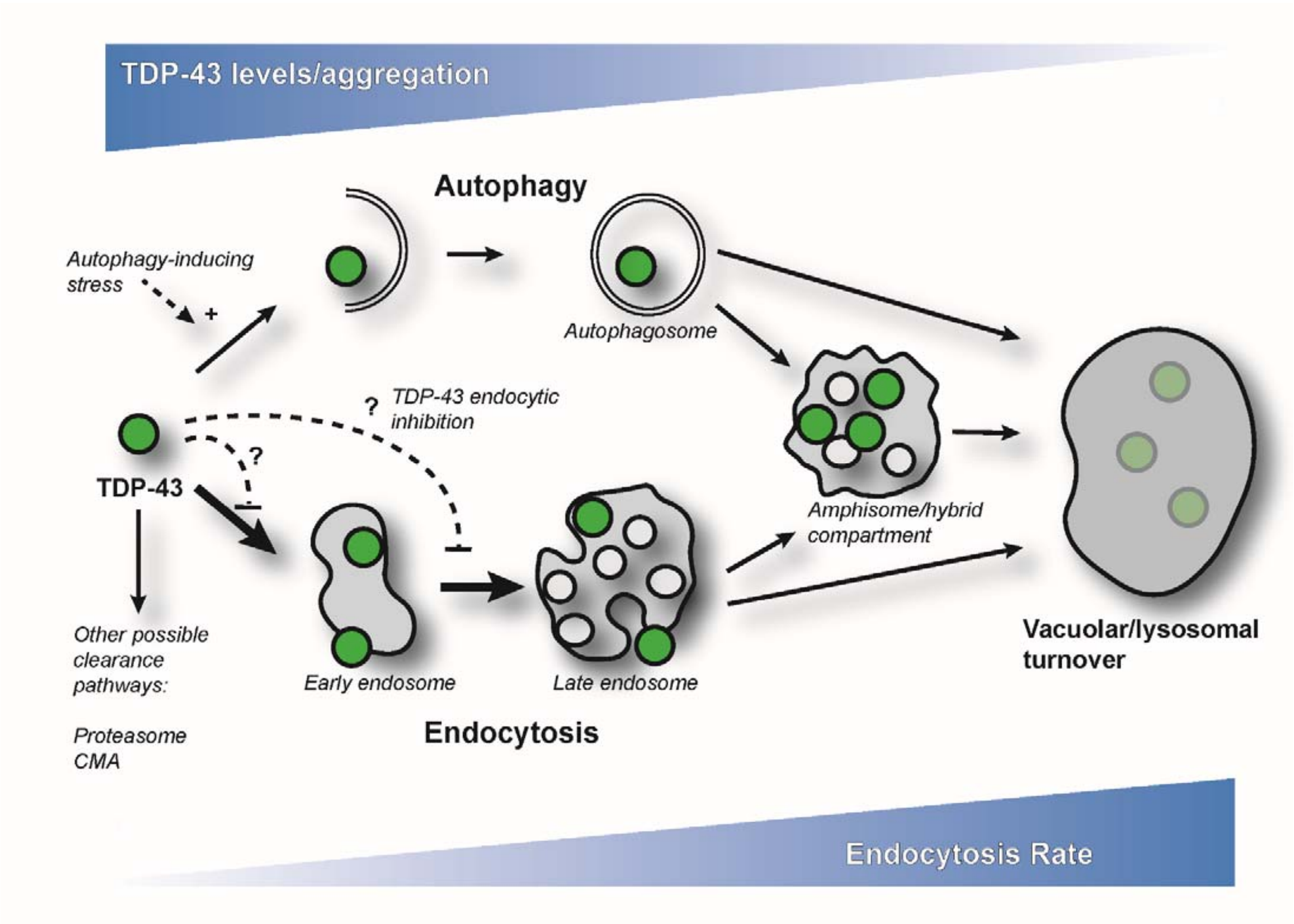

Figure S9: A possible model of endocytosis-dependent TDP-43 toxicity and turnover. TDP-43 is trafficked to vacuoles/lysosomes by an endocytosis-dependent mechanism. TDP-43 also inhibits endocytosis by an unclear mechanism (dashed lines and ?), in which TPD-43 aggregation and levels negatively correlated with endocytosis rates (blue triangles). Induction of non-specific autophagy, such as during stress, may enhance turnover of TDP-43 by autophagy. Convergence of endocytosis and autophagy in amphisome/hybrid compartments may also facilitate TDP-43 turnover. TDP-43 may also be cleared by Chaperone Mediated Autophagy (CMA) and proteasomal function in human cells.

Figure 1a

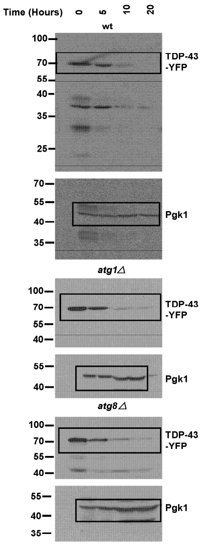

Figure 1b

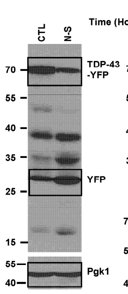

Figure 1c

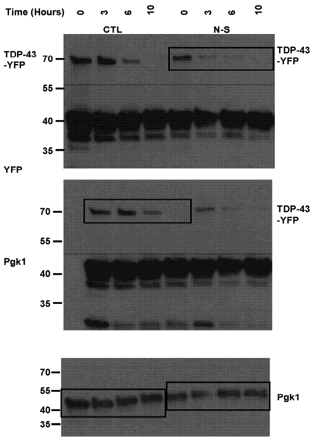

Figure 1e, Left

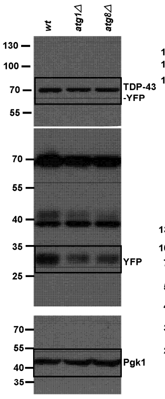

Figure 1e, Right

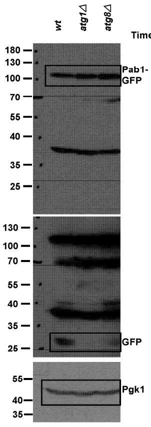

Figure 1f

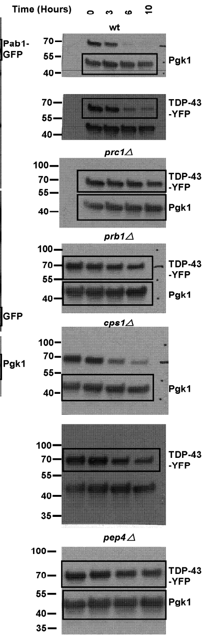

Figure S1b

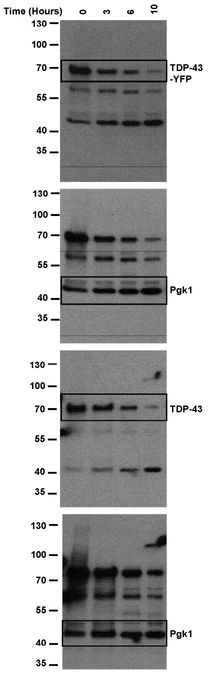

Figure S1c

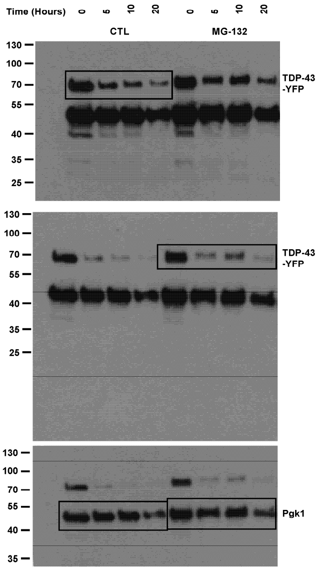

Figure S1d

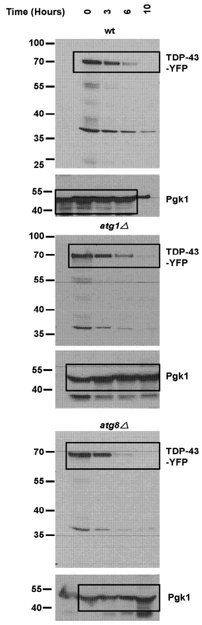

Figure S1k, Left

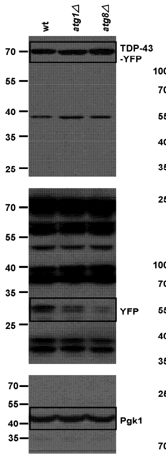

Figure S1k, Right

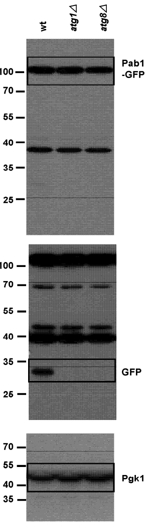

Figure 2b

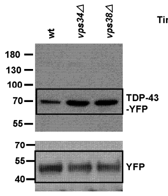

Figure 2d

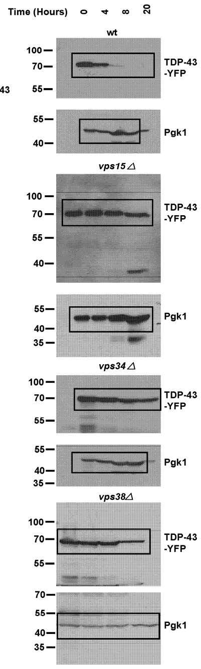

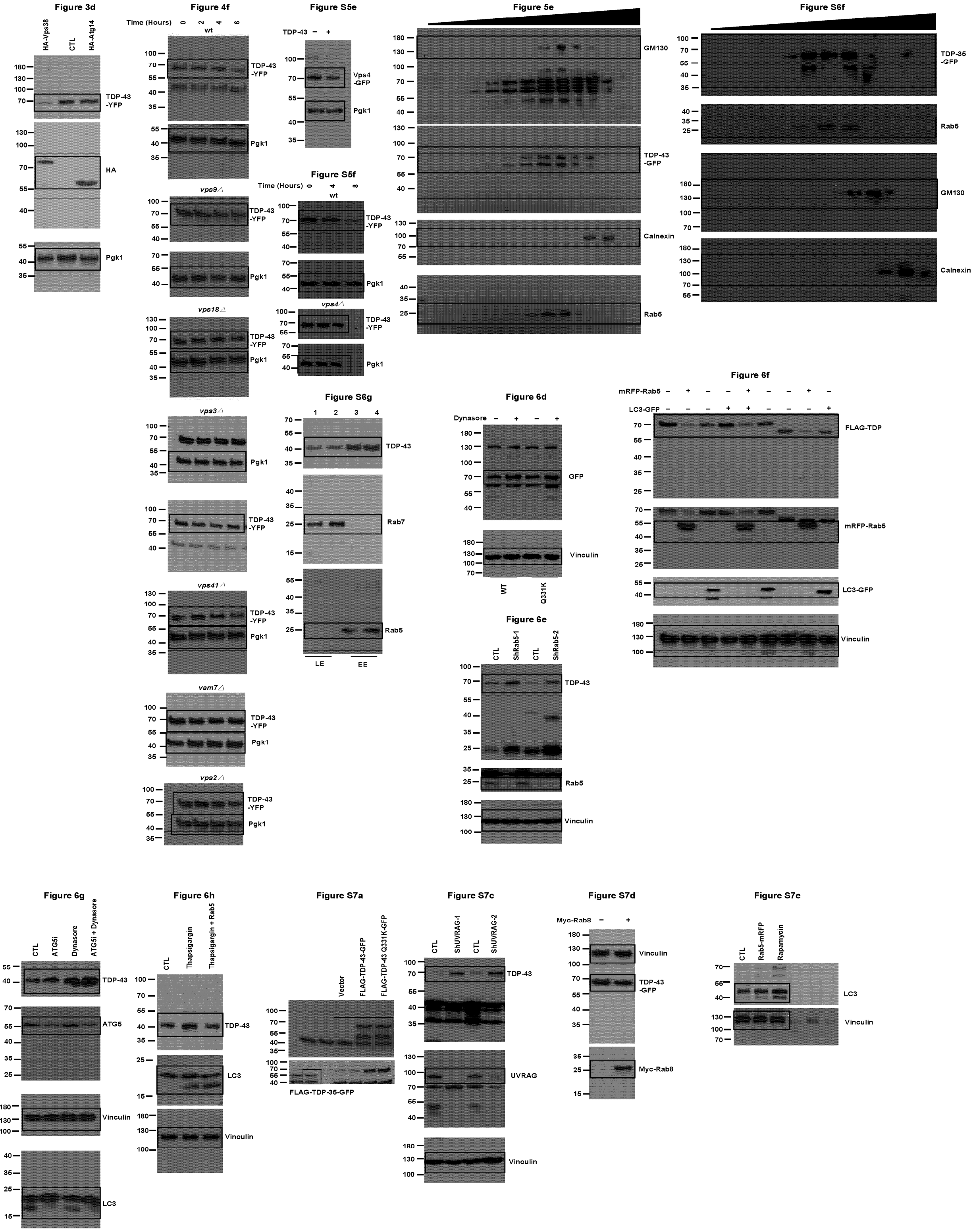

Figure S10: Uncropped Western blot images
