# Supplementary material for: Endocytosis regulates TDP-43 toxicity and turnover
Source: Nat Commun. 2017 Dec 12;8:2092. doi: 10.1038/s41467-017-02017-x (PMC5727062; doi:10.1038/s41467-017-02017-x)
Supplement: Supplementary file 3 — Description of Additional Supplementary Files [file 41467_2017_2017_MOESM3_ESM.pdf]

Description of Additional Supplementary Files

File Name: Supplementary Data 1
Description: Strains, Plasmids and Antibodies used in this study

File Name: Supplementary Data 2
Description: Microscopy Cell and Foci counts
